# Out of darkness: long non-coding RNAs come of age

**DOI:** 10.3389/fgene.2014.00388

**Published:** 2014-11-07

**Authors:** Yingqun Huang, Romano Regazzi, William C. Cho

**Affiliations:** ^1^Obstetrics, Gynecology, and Reproductive Sciences, Yale University School of MedicineNew Haven, CT, USA; ^2^Department of Fundamental Neurosciences, University of LausanneLausanne, Switzerland; ^3^Department of Clinical Oncology, Queen Elizabeth HospitalKowloon, Hong Kong

**Keywords:** lncRNAs, metabolism and obesity, neuronal disorder, beta-cell dysfunction, developmental cognitive neuroscience

It has been known for a number of years that only about 2% of this RNA encodes proteins. However, numerous studies employing both tiling arrays and high-throughput sequencing found that the genome is pervasively transcribed, with most DNA copied, at least at some point in time, into RNA. Indeed, Birney et al. (2007) estimated that 93% of the human genome is transcribed. Because of a dearth of functional information about such transcripts, the concept of widespread non-coding regions became the “dark matter” of the genome (Johnson et al., 2005) and in recent years there has been an explosion of research in this area. Due to technical and theoretical considerations, transcripts longer than 200 nucleotides and lacking the potential to be translated have been coined “long non-coding RNAs” or lncRNAs. Owing to thousands of these new transcripts that have been identified [The current GENCODE v20 estimates close to 15,000 independent lncRNAs in humans, much of the work laid on the discovery and characterization. Actually, some lncRNAs are very abundant and have been studied for many years (e.g., Xist RNA and H19 RNA), many of the others are expressed at much lower levels. Do they represent transcriptional noise? Are they often artifacts of sequencing? We are now emerging to get answers to these important questions. The past several years have witnessed striking progress in the functional characterization of many lncRNAs and a picture is now showing an enormously complex collection of transcripts, many of which are not at all inert, but rather play critical roles in cell function, gene regulation, and the development of disease (Morris and Mattick, 2014). Interestingly, lncRNAs can localize to the cytoplasm or nucleus, bind the proteins and other RNA molecules in mediating important intracellular interactions. Thus, among other functions, some have been shown to act as chromatin regulators, some influence transcription as enhancer-associated RNAs, some are host genes for smaller RNAs such as miRNAs and sno-RNAs and some act to sequester and modulate the function of miRNAs.

While much remains to be learned, we are truly at the frontier of important discoveries in the lncRNA field. The articles in this special issue continue this exciting trend connecting lncRNAs and cellular function, focusing particularly on their roles in development, metabolism, and association with the disease.

Several papers address the connection between lncRNAs and metabolism. Kameswaran and Kaestner (2014) discuss the growing evidence that lncRNAs can play an important role in the control of pancreatic beta-cell function and in diabetes manifestation. They particularly focus on lncRNAs generated from imprinted loci, where expression only occurs from either the maternal or paternal allele. Pullen and Rutter (2014) describe how genome-wide association studies have provided insights into ways in which lncRNAs can affect beta-cell identity and diabetes susceptibility. Esguerra and Eliasson (2014) describe the discovery and functional analysis of thousands of lncRNAs in the pancreatic islets of Langerhans and discuss how these transcripts might affect islet development and endocrine cell functions, and how understanding their biology might lead to therapeutic insights for the treatment of type 2 diabetes. In addition, Kornfeld and Bruning (2014) review the functional connection between lncRNAs, differentiation and homeostasis of metabolic tissues.

The role of lncRNAs in the nervous system are also addressed. Clark and Blackshaw (2014) and Vucicevic et al. (2014) review the current state of research on the emerging roles of lncRNAs in nervous system development and provide insights into how some of these might contribute to neurological pathologies. Kadakkuzha et al. (2014) contribute an original research article on the molecular characterization and functional analysis of the expression, localization and action of a lncRNA from the marine snail *Aplysia californica*, which is a natural antisense RNA from the sensorin gene and it plays an important role in neuronal function and aging.

Finally, there is an opinion article (Kohtz, 2014) by Kohtz underling the importance of interpreting the results with caution from studies on lncRNA function gleaned from cell culture model systems since they may not always accurately show us their natural *in vivo* functions.

## Conflict of interest statement

The authors declare that the research was conducted in the absence of any commercial or financial relationships that could be construed as a potential conflict of interest.

## References

[B1] Birney E. Stamatoyannopoulos J. A. Dutta A. Guigó R. Gingeras T. R. Margulies E. H.. (2007). Identification and analysis of functional elements in 1% of the human genome by the ENCODE pilot project. Nature 447, 799–816. 10.1038/nature05874 17571346 PMC2212820

[B2] Clark B. S. Blackshaw S. (2014). Long non-coding RNA-dependent transcriptional regulation in neuronal development and disease. Front. Genet. 5:164. 10.3389/fgene.2014.00164 24936207 PMC4047558

[B3] Esguerra J. L. Eliasson L. (2014). Functional implications of long non-coding RNAs in the pancreatic islets of Langerhans. Front. Genet. 5:209. 10.3389/fgene.2014.00209 25071836 PMC4083688

[B4] Johnson I. M. Edwards S. Shoemaker D. Schadt E. E. (2005). Dark matter in the genome: evidence of widespread transcription detected by microarray tiling experiments. Trends Genet. 21, 93–102. 10.1016/j.tig.2004.12.009 15661355

[B5] Kadakkuzha B. M. Liu X. A. Narvaez M. Kaye A. Akhmedov K. Puthanveettil S. V. (2014). Asymmetric localization of natural antisense RNA of neuropeptide sensorin in Aplysia sensory neurons during aging and activity. Front. Genet. 5:84. 10.3389/fgene.2014.00084 24795747 PMC4001032

[B6] Kameswaran V. Kaestner K. H. (2014). The Missing lnc(RNA) between the pancreatic beta-cell and diabetes. Front. Genet. 5:200. 10.3389/fgene.2014.00200 25071830 PMC4077016

[B7] Kohtz J. D. (2014). Long non-coding RNAs learn the importance of being *in vivo*. Front. Genet. 5:45. 10.3389/fgene.2014.00045 24624134 PMC3940894

[B8] Kornfeld J. W. Bruning J. C. (2014). Regulation of metabolism by long, non-coding RNAs. Front. Genet. 5:57. 10.3389/fgene.2014.00057 24723937 PMC3971185

[B9] Morris K. V. Mattick J. S. (2014). The rise of regulatory RNA. Nat. Rev. Genet. 15, 423–437. 10.1038/nrg3722 24776770 PMC4314111

[B10] Pullen T. J. Rutter G. A. (2014). Roles of lncRNAs in pancreatic beta cell identity and diabetes susceptibility. Front. Genet. 5:193. 10.3389/fgene.2014.00193 25071823 PMC4076741

[B11] Vucicevic D. Schrewe H. Orom U. A. (2014). Molecular mechanisms of long ncRNAs in neurological disorders. Front. Genet. 5:48. 10.3389/fgene.2014.00048 24624135 PMC3941653

